# The Week's Work

**Published:** 1909-10-02

**Authors:** 


### The Week's Work.

Last week the American Hospital Association held its eleventh annual congress at Washington. The session opened on Tuesday, September 21, when the delegates and members were welcomed by the President, Dr. John M. Peters, superintendent of the Rhode Island Hospital, who delivered an opening address on the present-day aspects of hospital work. At the evening session several important papers were read, among them being Mr. Sutton's "The Hospital and the Public," and Dr. Gilman Thompson's "Hospitals from the Patients' Point of View," both of which led to animated discussion. Dr. Sarason, of Berlin, also communicated a further report on his Terrace Pavilion System, to which we have already drawn attention in former issues. The system, which we understand is being considered by the authorities in the planning of the new wing of the London Homœopathic Hospital, provides for the proper lighting of balconies by direct overhead sunlight by shifting the balcony wall several feet to the rear, so as to produce a series of terraces instead of allowing the balconies completely to overlap one another.

On Wednesday, September 22, Dr. Brush, the able and energetic superintendent of the New York Post-Graduate Medical School and College, read a paper on "The Hospital and the Patient of Moderate Means"; Dr. Howell, superintendent of the New York Hospital, discussed "The Cost System of Hospitals"; while Mr. Homer Folks, the secretary of the American State Charities Aid Association, read a thoughtful and elucidative contribution on "The Many-sidedness of Hospital Work." The programme for the evening included "Suggestions on Hospital Construction," by Dr. Corwin; "Some Mexican Hospitals," by Mr. Taylor; and a paper on the newly-erected naval hospital, North Chicago, Ill., by Rear-Admiral Ross, superintendent. An attractive round of excursions, visits to representative institutions, and committee meetings filled the remaining days, and the Congress adjourned finally on Friday, September 24.

---

Commenting on the work of the Congress, the *International Hospital Record* remarks in its current issue that the Association has come to be one of the really representative organisations of the Western Continent. It is ten years old, and started in a small and unpretentious manner, having been founded in Cleveland in 1899 with a few members, who have enthusiastically worked to make it a success. "The records of sessions disclose the performance of volumes of important business details, improvements in hospital book-keeping, schemes of hospital architecture, points on dietetics, hospital sites, plans for larger economies, designs for the meek and lowly—nay, harmless—nurse, ideas for repressing the restless resident, conjectures as to the quality and quantity of spiritual pabulum that should be ladled out, the texture, colour, and textile strength of cottons and bandages, prices, and again prices—these have been some of the big and little things that have run the gauntlet of intellect and tongue in the years that have composed the strenuous decade of existence of this Association."

---

Dealing further with the trend of thought at these Congressional meetings, our contemporary proceeds: "The trend is consistently towards the patient, and boulevards now lead to him where a decade agone a dimly defined bridle path was thought large and up to date enough. . . . Some of us think thoughts that mean more than we have courage to give expression to, thoughts which if they had form and expression would revolutionise present-day management and give to those who need hospitals the atmosphere and—well, all that a hospital means. Ideals are still very far upon the air; architecture is still unsatisfactory, and its results are still unsafe; sites are not what they should be, and public interest is far from level with the details of development that are present-day property but are being ignored and neglected. Students of civics and correlative branches are giving more and more attention to the matter of the public sick and injured than at any other time, and this inquiry is sure to bear fruit. Out of it some day will come a spirit of independence, from which again will come an initiative and an individuality that will spring something millennial for the hospital."

---

A correspondent in a recent issue of the *Times* draws attention to one provision in Mr. Lloyd George's Finance Bill which "if unamended will undoubtedly impose a new tax on gifts to the nation." Hitherto a voluntary disposition of land, a gift, has been charged with a fixed stamp of 10s. The Finance Bill proposes to charge such a gift with the same stamp as though it were a conveyance on sale; that is to say, with 1 per cent. on the value of the land given. Land given for any charitable or public purpose will thus be charged in favour of the State with a hundredth part of its value, a charge which must be paid either by the donor or by the body which is to take charge of the gift. On the original foundation of a charity no doubt the tax will be paid by the founder; there will be no one else to pay it. But when gifts are made to existing institutions the expense will mostly fall on the public, as one can hardly look a gift-horse in the mouth or ask a public-spirited donor of valuable property to incur a substantial expense in addition.

---

According to the writer all other gifts of land for public or charitable purposes will be similarly affected by the Budget, and he proceeds: "Even when the charity is of the most restricted character the nation succeeds to a certain interest in the gift; for at any time the scope of the charity may be widened, and its objects more or less changed. If the general drift of present legislation is to increase the interest of the nation in its lands, it seems inconsistent to place impediments in the way of gifts for public purposes." The whole matter is one which is receiving the earnest consideration of governors and managers of charitable institutions all over the country. It is in the highest degree absurd that such institutions should be made to suffer in order to increase the funds available for old age pensions and other schemes of a less generally charitable character.

---

Mr. W. Towle, of the Midland Railway Hotels Department, has compiled a statement showing what would be the effect upon hotels and catering generally were the hotel clauses in the Finance Bill to pass unamended. In a covering letter, which we much regret that exigencies of space forbid our publishing in the columns of The Hospital, he draws attention to the German system of hotel taxation. It is striking to find that the total amount charged for local and imperial taxation on a large German hotel "does not exceed the amount which the Chancellor of the Exchequer, on the most favourable reading of his proposals, seeks to impose for license alone on the Queen's Hotel at Leeds." The net profits arising from the sale of liquor in a well-ordered hotel, after charging cost of the service and the upkeep of the rooms and places where it is served, he continues, is practically very little indeed, and the return on capital invested in hotels throughout the country does not average 4 per cent.

Some of the details he puts forward to strengthen his case will bear quotation as they are by no means generally known. Thus one thousand pounds of receipts for intoxicating liquor sold in hotels costs about seven times as much to distribute, owing to the amenities and expenses, as a similar amount costs in public-houses, and probably three times as much as in clubs. The larger number of servants necessarily employed in hotels, makes the consumption of beer and wine for the use of the staff much larger than can possibly be the case in a public-house or club, and consequently the poundage on purchases would have to be still further modified in the case of a hotel. It is perhaps not generally known that the wine used in every first-class hotel kitchen for staff consumption, sauces, etc., already pays to the State more than its actual cost price in duty alone. These statements are well worth consideration, and the Budget as a whole deserves the attention of everyone interested in institutional work. We hope to deal with its institutional aspects at some length in a future issue.

---

The Blue-book containing reports of and statistical information about the various Cape Colonial State-aided hospitals has just been issued by the Government of the Cape of Good Hope. At the end of 1908 there were 24 State-aided hospitals in the colony, all of which have made satisfactory progress. A reduction in the annual Government grants was rendered necessary owing to the generally felt financial depression, and an all-round 10 per cent. decrease was authorised. Most of the institutions, it is gratifying to notice, effected concomitant economies, the cost per patient being very much reduced in some instances. The average cost per patient for the 24 hospitals works out at 6s. 3d., or one penny less than in 1907, and sixpence less than in 1905. The lowest average cost per patient was shown by the Wynberg Hospital, where little more than a shilling per day per patient was spent. Against this is the eleven shillings per day per patient of two hospitals.

---

The general report goes on to deal, very briefly, with an important matter of hospital ethics. During the course of the year it appears that the constitutions and rules of several hospitals were completely revised, and it is pleasing to record that in each case a regulation has been included providing that no patient shall pay fees to his private medical attendant until the full charges of the hospital have been defrayed. It is difficult to understand why such strenuous opposition to the introduction of this principle—one which appears to be so necessary and serves so useful a purpose—has been met with in the past from various Hospital Boards, although when actually applied it has always been found to operate satisfactorily. As it was noticed that in certain instances members of Hospital Boards were at the same time contractors for supplies or services to the hospital a circular was issued suggesting the adoption of the following regulation:—"No member of the Board of Management shall, either directly or indirectly, be concerned in or participate in the profit of any contract with the hospital, or be concerned in or in the profit of any work to be done in connection with the hospital." Considerable correspondence resulted and many of the boards were at first strongly opposed to the suggestion. The regulation has now, however, been adopted verbatim by nearly all the hospitals. In a few instances the matter is still under consideration, but there is reason to believe that the regulation will shortly be in operation in all the institutions.

